# Supplementary material for: Patterns of Intron Gain and Loss in Fungi
Source: PLoS Biol. 2004 Nov 30;2(12):e422. doi: 10.1371/journal.pbio.0020422 (PMC532390; doi:10.1371/journal.pbio.0020422)
Supplement: Table S1 — Also available at http://genes.mit.edu/NielsenEtAl/. (4.3 MB ZIP). [file pbio.0020422.st001.zip › NielsenEtAl/html/1399.html]

AN7677.1.NCU06637.1.MG00759.1.FG10096.1


```
 CLUSTAL W (1.82) Multiple Sequence Alignments - Introns Inserted


Sequence 1: NCU06637.1	529 aa
Sequence 2: FG10096.1	501 aa
Sequence 3: MG00759.1	610 aa
Sequence 4: AN7677.1	517 aa
Alignment Length: 703 aa
Number Identitical Residues: 156 aa
Alignment Score (without introns) 8683


MG00759.1 	MIHVSTLVNVFIKGQV1YDLAWLGFCTKCARLYGVRKIIVSEVQASTLFVDCDKI1HFES
NCU06637.1	----------------~--------------------------------------~----
FG10096.1 	----------------~--------------------------------------~----
AN7677.1  	----------------~--------------------------------------~----
          	                                                            

MG00759.1 	WVDRVFTFQTTNLGWLVRITSRNKQLSEMDELSSHVLTQTNPSTPATSAADYRDDE----
NCU06637.1	----------------------------MDDLAAAFLGQGDDASIQEEDDGQQGSG----
FG10096.1 	----------------------------MDDFTAEMFATGRGSDSAEDSRRRRGHGDDDD
AN7677.1  	----------------------------MQEYTPGAFADRDDPTPP-----LRGRQ----
          	                            *:: :.  :     .         :.      

MG00759.1 	-------------VLEGGDGSPNKDKKRRSLRNFLKGGADMQDKLLEK2LLQQIIPSDLD
NCU06637.1	----------------SGTDSTTTARTLRQG---ILKAANLQDKLLEK2LLAQVIPVD-D
FG10096.1 	KTPATGADSNSPSNLPSGSDTPPQKTGLRGGFAAIRQKANIQDRLVEK2LLQQVIPTN-D
AN7677.1  	------------------TASTDDQISSRHERSVSSIRQSMQDRLLTK2ILEQVVPVE--
          	                    :.      *          .:**:*: * :* *::* :  

MG00759.1 	SAGGGLSFTGDG----SPYSRPAFSLPTMSYNFRRFNTR~IGVVFKGQIKLERLVTWRHP
NCU06637.1	GQPHGSGEPGGANEPSAFGERPGFTVGAMSNNFRRFNAR~IGVVFKFQAKALRVLSWRRP
FG10096.1 	DDQNDVHFVGDE-QTATQTERPNFNITTMSFNFRRFNAR~IGVVFKFQARVERILSWKQA
AN7677.1  	-DVHDESVSVGAKPVPPDAQRPAFSLPLMGNNFRRFNAR2IGIVFHFQEQVERLFAWKQP
          	    .     . .  ..  .** *.:  *. ******:* **:**: * :  *:.:*::.

MG00759.1 	TRTLSFLAVYTLVCLDPYLLSVLPMVLLLTAVLVPSFIARHPDPPSAGSAPGLASGGGVS
NCU06637.1	THTLSLLAVYTFVCLDPYLIFVLPLAIALFGIFVPSFIARHPAPASSSEAHAVRNLG---
FG10096.1 	SHTLSLLAVYSFVCLDPYLIFVLPIAILLLGVFIPSFMARHPAPPKGT-LSSEQNVG---
AN7677.1  	SHTFSFLFVYSFICLDPHLLVVLPIATILLFVMVPTFLARHPPPPSTSTSSIT-------
          	::*:*:* **:::****:*: ***:.  *  :::*:*:**** *..              

MG00759.1 	GTGANAASVHADDAARTRSYSSKQHTTYNPRGPPVAPARTVKPVKELSRDFFRNLGHLQN
NCU06637.1	---------------------------YSPRGPPLAPARTFKPTKELSRDFFRNLGDLQN
FG10096.1 	---------------------------YSPQGPPLAPAATVKPVKELSKDFFRNMRDLQN
AN7677.1  	-------------------------PYYSYQGPALAPPKTIKPAPETSKDFFRNMRDLQN
          	                         . *. :**.:**. *.**. * *:*****: .***

MG00759.1 	TMADFVTAHDAIVETMVPATNFSDEARSSALFVALFFASAVMSIAAHLLPWRLLLLVGGW
NCU06637.1	VMDDYSVAHDKIIALVVPKTNFSDEALSSAIFVLLCGACALMTVTAHLIPWRYICLVAGW
FG10096.1 	CMDDFSRGHDQVVALMVPVTNFSDEALSSTLFLFLTVGGIAMTVSAHLLPWRIIFLVGGW
AN7677.1  	CMADFSDLHDSAVSALAPMTNFSNEKLSSAVFLSCTVLTAVLFLTAHLIPWRYIMLIGGN
          	 * *:   **  :  :.* ****:*  **::*:        : ::***:*** : *:.* 

MG00759.1 	AALISGHPAVHRRAAAYAAR--GRPKTEMTRAKTLLER----------------------
NCU06637.1	LAISSNHPTVSRLIGEWQAQYLGGEKEPSATAQAVHDNDKKLEQIQNHPPGPSPDSSLAA
FG10096.1 	AIVGMGHPHVARLVTAAHREHLQPQE---AKAQSWLEN----------------------
AN7677.1  	AALLSKHPNIQEFLQGLAAELGSEELTQVPSGKAPAPHMP--------------------
          	  :   ** : .       .         . .::   .                      

MG00759.1 	------FVDADIILDQAPETREVEIFELQRRNSGD--ADEWEPWKFSPTPYDPMSPSRLG
NCU06637.1	ERAFDAWVRSDIILDDAPETREVEIFELQRLSDT---SGEWEPWLFSPSPYDPLSAARIA
FG10096.1 	------WTSSDIILDTSPETREVEIFELHRPTSG---GGEWEPWLFCPSPYDPLSQPRIA
AN7677.1  	----FMKLLDDISLDSYPEEREVEIFEIQYRSLAPYAEQDWETFLFSAVPYDPLSPSRIA
          	          ** **  ** *******::  .    :  :**.: *.. ****:* .*:.

MG00759.1 	GGNPPQGARYFEDVLPPEGWEWSEKKWALDLFSREWVEEGIITGVEVETEGE--------
NCU06637.1	G-DRPTGTRFFEDVLPPEGWEWSGKKWALDLWSREWVEERIITGVEVETEGE--------
FG10096.1 	G-ERPRGSRFFEDVMPPEAWEWSEKKWALDLWSREWVEERIITGVEVETEGE--------
AN7677.1  	G-DRPKGCRFFEDVQPPAGWAWKSKKWELDLDCREWVVERMITGVGFEVPGSPSESGAGS
          	* : * * *:**** ** .* *. *** *** .**** * :**** .*. *..:.:.:.:

MG00759.1 	----RWVYDMYNENEG--------------VTGVVEQPISSSDM---ARKGKGHTRTMTP
NCU06637.1	----RWVYDIYNEHENGVNGSDNGDLGAALGDGDYETPVRS--------QDQSRYTPKIG
FG10096.1 	----RWVYDIWDERDE--------------RTGVVDLPVND--------KGKQKATPRP-
AN7677.1  	DEIGGWVWDLPSASSLR-------------SDDDVASALGYGTLGQIGEKKKGKKKGKER
          	.. . **:*: .  .                 .    .:  .  .. . : : :      

MG00759.1 	STVSWEE-GFEGVGKRGEWRRRRWTRLVRRKKFAGTA----------
NCU06637.1	PTISWEE-GEEGMGRRGSWRRRRWVRMVKRKNVNAASN---------
FG10096.1 	---SWEE-NEDGNGRRGNWRRRRWVRLVKRKTIPLQPN---------
AN7677.1  	ATQDYEEKGSPGSNVMGEWRRRRWVRIVQRISMPAEKEKAKSRGNNA
          	.: .:**..  * .  *.******.*:*:* ..    ..:.: ...:
```
